# Proceedings of Veterinary Societies

**Published:** 1889-04

**Authors:** 


### PROCEEDINGS OF VETERINARY SOCIETIES.

---

The Twenty-sixth Semi-annual Meeting of the United States Veterinary Medical Association was held at Young's Hotel, Boston, March 19, 1889. President Huidekoper called the meeting to order at 11 A. M. On roll call the following members responded to their names: Drs. Bailey, Burns, Thomas Bland, Blackwood, Breakell, Bryden, Clement, Cochran, Faust, Gill, Hugg, C. H. Hall, Harrison, Hawk, Hoskins, Howard, Huntington, Huidekoper, Hitchcock, Liautard, Martinet, W. B. E. Miller, L. McLean, R. A. McLean, Mustoe, Osgood, Peabody, Peters, J. L. Robertson, A. H. Rose, Ross, T. B. Rayner, James B. Rayner, J. S. Saunders, R. J. Saunders, J. H. Stickney, Simmonds, Sherman, Tuttle, Winchester, Chas. Winston and Zuill. Drs. Russell, of Maine, W. T. Russell, of New Hampshire, and Bronnell, of Massachusetts, were present as applicants for membership.

The minutes of the annual meeting were read and adopted. The report of the Comitia Minora was then read and adopted, directing a revision of the Constitution and By-laws. Drs. Hinckley and Lusson were recommended for membership and afterwards elected. The application of Dr. Edgar R. Martin, of Philadelphia, was rejected on the grounds of violation of Code of Ethics, in holding a contract under Live Stock Insurance Company to the detriment and injury of his fellow-members.

The following resolution, offered by Dr. Huidekoper, was adopted:

*Whereas*, The Comitia Minora find that many of the By-laws of this Association have been violated, that candidates elected have attended meetings without payment of dues or signing the Constitution, that members have been dropped from the rolls without notice being given to the Association; therefore

*Resolved*, That the committee appointed to revise the Constitution and By-laws consider if names exist on the rolls of the Association which are not entitled to membership, and report the same at the next meeting.

The recommendation of the Comitia Minora to hold the next annual meeting of the Association in September in Brooklyn was adopted.

The Committee on Intelligence and Education, through the chairman, Dr. Coates, made an interesting report of the various veterinary movements during the preceding six months.

The chairman, Dr. Liautard, made a brief report of the lack of success of the Committee on Army Legislation.

Under the head of new business Dr. Liautard offered the following resolution:

*Resolved*, That this Association shall hereafter hold only one meeting yearly, on the third Tuesday of September, and that the officers of the Association make the necessary arrangements to have said meeting last at least two days.

On being seconded it was laid over until the next meeting of the Association.

The afternoon session convened at 1.30 P. M., when the Chairman asked for the reading of essays.

Dr. Winchester responded with one on the subject of "Tuberculosis," giving in interesting detail the history, mode of development and dangers of the ingestion of food and flesh from these tubercular subjects. In his excellently prepared article he quoted leading authorities abroad and at home, showing the danger and means of transmission from animal to man.

The reading of the paper was followed by remarks from Dr. Miller, of New Jersey, showing its prevalence in his State and the rapid increase it was making, with no laws or safeguards to delay its progress and dangers.

Its existence in New York State was referred to by Dr. J. Faust, who specially quoted an incident of its rapid destruction of the usefulness of a herd of thoroughbreds, likewise referring to the existence of laws in the seventeenth century against selling the milk or using the flesh in Germany, and contrasting the absence of such safeguards now.

Dr. Liautard, in a few well chosen remarks, called for some means of arousing public sentiment to the importance and grave dangers of this disease, and suggested that veterinarians should in their States and respective localities use more freely the channels of the public press to aid them in at least limiting its progress and lessening its evils.

Dr. L. McLean, of Brooklyn, reasoning from the admitted and undoubted facts of its transmission from animal to man and the recognized gravity of the danger by the veterinarian, suggested that if the milk cans from some of the herds infected with tuberculosis were labeled "Consumption at eight cents per quart" it would not be putting it too strong, and would probably arouse the people from their state of lethargy. He then painted a word picture of the head of a family afflicted with consumption, to whom only an overcrowded hospital for such cases could be recommended, while the fruitful cause of this sad affliction remained undisturbed and unguarded.

Dr. Peabody of Rhode Island: Perhaps what I have to say would not be of much interest to us, but I want to say a few words, as far as my knowledge goes, but I have had a case come under my notice, which is of interest to every member of the Association.

Some eight or nine years ago I diagnosed a case of tuberculosis; the cow had been purchased to supply milk to a child whose mother had died in child-birth, and the child was not doing well; I made the diagnosis, as I have said, and found the cow suffering from tuberculosis. The gentleman who had purchased the cow said he had bought it on purpose to supply the child with milk; I told him what was the matter with the cow and that it should not be allowed to live. The remark he made was, "Well, I paid $125 for her, and I guess you have got it down too fine." The cow kept growing poor; that was in April; the next May it died and I made a post-mortem examination and found the lungs, spleen, and in fact every organ of the body was filled with tubercles. The next August the child died, and the physician who made the post-mortem examination of the child found that it died of meningical tuberculosis. No one had known before that time of any tendency of that kind in that family; two years after, another child, older, died, and last year another one of the family died of tuberculosis. Whether it all came from that cow I can't say; but they had another cow in the barn that had tuberculosis, and I killed it; that was to me a direct case of inoculation.

When I was with Dr. Liautard in the hospitals in making post-mortems on the animals that came from Central Park, we fed some kittens with some of the meat from those animals, and they died of tuberculosis. For fear of being laughed at, and being, as the Dr. says, "bashful," I said nothing about it, but I followed the matter up and studied it up, and in Rhode Island, within a radius of five miles I fear that not a herd is there but what I can find some tuberculosis in. My friend, Dr. Stickney, too, found some of them; thirty were killed and not one of them but were more or less suffering from tuberculosis.

Further remarks were made by Mr. Stockbridge of the Massachusetts Cattle Commission, verifying many of the statements made by the essayist and reporting recommendations made to the State on the subject.

The subject was further discussed by Mr. Sessions of Massachusetts, and several others after which a vote of thanks was tendered to the essayist.

The President then announced the following Committee on Revision of the Constitution and By-Laws: Dr. Miller of New Jersey, Dr. Goentner of Pennsylvania, Dr. Clement of Maryland, Dr. Harger of Pennsylvania, and Dr. Robertson of New York, with the President and Secretary members *ex-officio.*

Dr. Liautard then read an article entitled "Recording Clinical Observations." It proved to be one of the most suggestive papers read before the Association. The advice and suggestions it contained were fraught with great value to the coming generation of veterinarians, if followed by the present, in building up through the channels of records of clinical diseases, a literature for the Veterinary World that would broaden and increase its value to an inestimable extent.

After some discussion, Dr. Gadsden's paper on "Mediate Contagion of Contagious Pleuro Pneumonia, was read by the Secretary and the discussion of the same was on motion postponed to the annual meeting.

The President called attention to the International Medical Congress, to be held in Paris, the coming Summer, and suggested that credentials of this Association be furnished any members who expect to be in Europe at this period.

The meeting then adjourned to meet in Brooklyn September 17th, 1889.

The evening was spent around the banquet table where forty-three members partook of the bountiful provision of good things so well prepared by the local Boston committee, and the day's enjoyable festivities and renewing of fraternal relations were completed by responses to the following toasts; "The Veterinary Profesrion in the U. S.," Prof. A. Liautard; "The Veterinary Profession in Canada," Dr. Bryden; "The Medical Profession," Dr. Ernst; "The Relation of Veterinary Medicine to Sanitary Science," Dr. L. McLean; "Agriculture," Hon. W. R. Sessions; "Relation of the Veterinarian to the Agriculturist," Hon. Levi Stockbridge; "The Press," Dr. Huidekoper; "The Humanitarian part of the Veterinarian," Col. Currier; "The early days of the Association," Dr. J. H. Stickney; "The Ladies," Dr. J. Faust; and several other toasts of a more local character by members of the profession.

W. HORACE HOSKINS,
*Secretary.*

THE ANNUAL MEETING OF THE PENNSYLVANIA STATE VETERINARY MEDICAL ASSOCIATION was called to order by the president, Dr. Thomas B. Raynor, March 5th, at 10.30 A. M., in the lecture room of the Veterinary Department of the University of Pennsylvania.

The following members responded to the roll, called by the Secretary: Drs. Thomas B. Raynor, W. L. Zuill, Alexander Glass, T. C. Michener, W. H. Hoskins, J. B. Raynor, W. S. Kooker, George B. Raynor, J. R. Hart, C. J. Blank, S. E. Weber, W. B. George, Z. S, Keil, J. W. Sallada, Charles F. Goentner and S. J. J. Harger.

The minutes of the last regular, as well as of the last special meeting, held December 13, 1888, were read and, with a single alteration, approved.

The election of officers to serve during the ensuing year resulted as follows: President, W. L. Zuill; First Vice-president, J. B. Raynor; Second Vice-president, Charles F. Goentner; Third Vice-president, T. C. Michener; Recording Secretary, S. J. J. Harger; Corresponding Secretary, W. S. Kooker; Treasurer, John R. Hart; Board of Trustees, W. H. Hoskins, chairman, T. C. Michener, S. J. J. Harger, C. J. Blank and Alexander Glass.

The following gentlemen applied for membership: W. B. Montgomery, V.M.D.; James Graham, M.R.C.V.S.; Robert Formad, V.M.D.; W. R. Ridge, V.M.D.; M. W. Broadhead, D.V.S.; J. F. Rose, V.S.; R. G. Webster, V.M.D.; A. T. Schreiber, V.M.D.; J. S. Luitsman, V.M.D.; Antoni Maurise, V.M.D.; B. F. Bachman, V.M.D., and R. A. Hummel, V.S. All of the applicants were elected as members, excepting Dr. Hummel, who failed to appear before the Board of Censors.

Dr. Hoskins, chairman of the Committee on Legislation, gave a verbal report of the progress of the bill to regulate the title of Veterinary Surgeon now before the Legislature. The bill, he said, was under favorable consideration, and would doubtless before long become a law.

Dr. Zuill, chairman of the Committee on Contagious Diseases, Sanitary Science and Police, submitted his report of the extent of contagious and infectious diseases, and formulated the work to be done in the future. Among other things he mentioned that the State was very nearly rid of contagious pleuro-pneumonia, and dwelt upon the prevalence of glanders and tuberculosis, and the methods to eradicate them. It was particularly urged that a veterinarian should be included in a Board of Public Health.

Dr. Hoskins, chairman of the Committee on Intelligence and Education, made a lengthy and able report in which he reviewed the history of the profession in the State and the rapid progress which it has made within the last ten years. He also spoke of its needs, defects, and future prospects, as well as of the dangers to which man and animals are exposed, the result of insufficient education and authority on the part of the veterinarian. Both these reports will be published for distribution.

An amendment to the constitution was offered, changing the members of the Committee on Sanitary Science and Police from three to seven.

The Secretary read letters of regret from Drs. Night and Gladfelter, and other extensive communications pertaining to legislation.

After a collation, which was served in the building by the Keystone Veterinary Medical Association, essays were read by Dr. Sallada on "Cremation," and Dr. Keil on "Overchecking." The discussion which followed called forth the opinion of many of the members. Dr. L. McLean, of Brooklyn, who was an invited guest, participated in the same. The latter paper excited a great diversity of opinion founded on practical observation. Dr. Sallada promised that at a future meeting he would continue the subject of cremation as applied to the lower animals.

The Treasurer's account showed a healthy surplus.

The next place of meeting will be Pottsville, and a large and interesting meeting is anticipated. Not only the Veterinarian but the human practitioner is invited.

S. J. J. HARGER,
*Secretary.*

---

THE NEW JERSEY STATE VETERINARY SOCIETY held its regular meeting at the Hamilton House, Patterson, on Thursday, February 7th, 1889, at 2 P. M.

Dr. A. T. Sellers, of Camden, a graduate of the American Veterinary College, was proposed for membership of the society, and it was referred to the Board of Censors, who will report at the next meeting.

The Secretary read Senate Bill, No. 12, to Regulate the Practice of Veterinary Medicine and Surgery, which is now before the legislature. A very lively discussion followed, after which the members present approved the bill. Dr. Lowe thought that the proposed bill could be enacted as a law if the veterinarians would unite to concerted action.

The delegates appointed to attend the meeting of the Alumni of the American Veterinary College are as follows: Dr. J. C. Corliss, Dr. W. H. Lowe, Dr A. H. McIntosh, Dr. J. F. Autenrieth and Dr. C. Kuehne.

The paper read by Dr. J. F. Autenreith on "The Operation of Tenotomy, its Application in Sprung Knee and Knuckling," was a very able one, and brought forth some very interesting facts. The author spoke highly of the operation in both of these diseases, the discussion following was very instructive.

Dr. W. H. Lowe made a motion to make an amendment to Article V of the Constitution which reads: "There shall be four regular meetings of this association every year; one in August, one in November, one in February, and one in May. Each meeting shall be held on the first Thursday of the respective months, with a banquet once a year at the annual meeting in August," to have bnt two meetings each year, the annual meeting to be held the first Thursday in August, and the regular meeting to be held on the first Thursday in February. The matter was submitted to the Board of Trustees, who will report at the next meeting.

It was decided to hold the next meeting in Newark, on Thursday May 2d, at 2.30 P. M., and the Board of Trustees to meet at 2 P. M.

The meeting then adjourned.

CHARLES KUEHNE, D. V. S.,
*Secretary.*

---

AMERICAN VETERINARY COLLEGE.—The Fourteenth Annual Commencement of this College was held March 4th, 1889, at Chickering Hall, New York City. C. A. Dormeus, M. D., awarded the prizes and A. J. Thompson, D. V. S., of the Graduating Class, delivered the valedictory.

Prof. F. D. Weisse conferred the degree of D. V. S. on the following graduates :—

Frank Allen, St. Paul, Minn. ; Harry E. Bates, New Haven, Conn. ; Waldo H. Brownell, New Bedford, Mass. ; Lewis J. Brodhead, Stone Ridge, N. Y. ; George W. Buckner, Liberty Centre, Ind. ; Theodore A. Burnett, Springfield Ohio ; Edwin T. Campbell, Tarrytown, N. Y. ; Samnel M. Campbell, Pottstown, Pa. ; Josiah C. Case, Peconic, N. Y. ; Matthew H. Cochran, New York, N. Y. ; William F. Doyle, New York, N. Y. ; Harry O. Driscoll, New Haven, Conn. ; Robert W. Ellis, Paterson, N. J. ; Gulian C. Fagan, New York, N. Y. ; William D. Farnum, Waltham, Mass. ; Frederick D. Greely, New Bedford, Mass. ; Gerald E. Griffin, Fort Robinson, Neb. ; Charles Hall, Randolph, N. Y. ; Walter L. Hart, Philadelphia, Pa. ; Harry D. Hanson, New York, N. Y. ; Herman Hausser, Jersey City, N. J. ; John Hooker, New Baltimore, Mich. ; John A. Huhne, Kingston, N. Y. ; Charles Jameson, Brooklyn, N. Y. ; George A. Jarman, Chesterville, Md. ; Edward F. C. Joerg, Oleona, Pa. ; James Johnstone, Dobbs Ferry, N. Y. ; John T. Lee, M.D., Minneapolis, Minn. ; Robert L. Leis, Newark, N.J. ; Richard R. Letts, Hoboken, N.J. ; Charles D. MacMurdo, Charlottesville, Va. ; William F. Mellows, Sing Sing, N. Y. ; Harry Morris, Providence, R. I. ; Richard R. Morrison, Sidney, Australia ; Nicolas L. J. Nicolas, Sarreguemines, France, Morice E. O'Connor, Denver, Colo. ; Joseph Ogle, Jr., New York, N. Y. ; Thomas S. Partlan, Rondout, N. Y. ; Clarence P. Stanbrough, Newburgh, N. Y. ; Harry B. Summy, Manheim, Pa. ; Martin J. Tewey, Irvington, N. Y. ; Albert J. Thompson, Lebanon, Ohio ; George G. Vandeveer, Brooklyn, N. Y.

---

The New York College of Veterinary Surgeons and School of Comparative Medicine held its thirty-second annual commencement at Steinway Hall March 13, 1889. The stage was occupied by the members of the Faculty and the resident Alumni of the college. The secretary of the Faculty then announced to President W. T. White the names of those recommended for the degree of "V. S." : Port Crego, George G. Pearson, H. Van Der Rost, Frank I. Winant, Frank C. Herbert, W. H. Robinson, Charles H. Perry, R. E. Stanwood, William H. Kelly, T. E. Maloney, William F. Walsh, U. G. Grimshaw, R. T. Churchill, E. M. Osborne, William A. Birch, William Appel, S. W. Matthues, C. J. Waidner, Robert Richards, John F. Page, Charles A. Mackey. The prizes were awarded by the president, in accordance with the decisions of the faculty, to William Frederic Walsh, gold medal ; Robert Richards, silver medal and best junior examination prize for 1888 ; George S. Fuller, McLean silver medal for best junior examination in 1889, and P. A. Davidson, Jenkin's prize for second junior examination in 1889. Frank I. Winant, V. S., delivered the valedictory address.

